# Moderate Physical Activity as a Prevention Method for Knee Osteoarthritis and the Role of Synoviocytes as Biological Key

**DOI:** 10.3390/ijms20030511

**Published:** 2019-01-25

**Authors:** Paola Castrogiovanni, Michelino Di Rosa, Silvia Ravalli, Alessandro Castorina, Claudia Guglielmino, Rosa Imbesi, Michele Vecchio, Filippo Drago, Marta Anna Szychlinska, Giuseppe Musumeci

**Affiliations:** 1 Department of Biomedical and Biotechnological Sciences, Human Anatomy and Histology Section, School of Medicine, University of Catania, Via S. Sofia n°87, 95124 Catania, Italy; pacastro@unict.it (P.C.); mdirosa@unict.it (M.D.R.); silviaravalli@gmail.com (S.R.); claudiaguglielmino11@gmail.com (C.G.); roimbesi@unict.it (R.I.); marta.sz@hotmail.it (M.A.S.); 2 School of Life Sciences, Faculty of Science, University of Technology Sydney, P.O. Box 123, Broadway, Sydney, NSW 2007, Australia; Alessandro.Castorina@uts.edu.au; 3 Discipline of Anatomy and Histology, School of Medical Sciences, The University of Sydney, Sydney, NSW 2006, Australia; 4 Department of Biomedical and Biotechnological Sciences, Section of Pharmacology, University of Catania, via S. Sofia 67, 95123 Catania, Italy; michele_vecchio@yahoo.com (M.V.); fdrago@unict.it (F.D.); 5 School of the Sport of the Italian National Olympic Committee “CONI” Sicily, Via Emanuele Notarbartolo, 90141 Palermo, Italy

**Keywords:** osteoarthritis, synovium, physical activity, interleukins, lubricin

## Abstract

The purpose of this study was to investigate the influence of moderate physical activity (MPA) on the expression of osteoarthritis (OA)-related (IL-1β, IL-6, TNF-α, MMP-13) and anti-inflammatory and chondroprotective (IL-4, IL-10, lubricin) biomarkers in the synovium of an OA-induced rat model. A total of 32 rats were divided into four groups: Control rats (Group 1); rats performing MPA (Group 2); anterior cruciate ligament transection (ACLT)-rats with OA (Group 3); and, ACLT-rats performing MPA (Group 4). Analyses were performed using Hematoxylin & Eosin (H&E) staining, histomorphometry and immunohistochemistry. In Group 3, OA biomarkers were significantly increased, whereas, IL-4, IL-10, and lubricin were significantly lower than in the other experimental groups. We hypothesize that MPA might partake in rescuing type B synoviocyte dysfunction at the early stages of OA, delaying the progression of the disease.

## 1. Introduction

Physical inactivity represents the fourth leading cause of morbidity and mortality globally. Regular exercise is highly beneficial for the management of many common chronic diseases [1]. There is substantial evidence for the benefits arising from physical activity in cardiovascular diseases, diabetes, obesity, and musculoskeletal conditions, including knee osteoarthritis (OA) [2]. Walking and running provide repeated loading to the knee, which is thought to be essential for joint’s health within a physiological window. Physical activity, as a fast track rehab, also becomes an important treatment in patients after arthroplasty, reducing hospitalization and recovery times [3]. However, exercising outside the physiological window, e.g. excessive cyclical loading, may produce mechanical stress that could be detrimental to joint health and lead to injury and, ultimately, to OA onset [1,2,3,4].

It is well known that movement requires positioning of the body weight in horizontal (running and walking) and vertical (jumping) directions, being managed by the knee joint, one of the major and most complex joints in our body [5]. The knee consists of a modified hinge joint, a type of synovial one, bathed in synovial fluid, which is contained inside the articular capsule that is coated by the synovial membrane. The synovium is a specialized connective tissue that envelops diarthrodial joints and maintains the fluid-filled cavity to provide a lubricating environment for the articulating surfaces [6,7]. Since the articular cartilage is avascular, chondrocytes extract the oxygen and nourishing factors that are needed for cell survival from the synovial fluid by simple diffusion. The synovium produces specific substances that lubricate the joint and paracrine factors, having an important impact on articular cartilage metabolism [6,7]. The synovium displays a superficial cellular lining that is composed of two types of synoviocytes: Type A cells containing vacuoles that are related to phagocytic function (macrophages) and Type B cells with secretory fibroblast-like functions producing hyaluronan and lubricin. The latter are the lubricating molecules embedded within an extracellular matrix, rich in collagen and proteoglycans that act as a semi-permeable membrane to mediate solute transport. The Type B cells are capable of transforming into fibrocytes, depending on the inflammatory response to coexistent cytokines [8,9,10]. Synovium has various purposes, including lubrication, phagocytosis, and immune function. Synovial lubrication diminishes the joint frictional coefficient in healthy joints, thus reducing heating and wearing of the cartilage [11]. The loss of synovial lubricating ability is implicated in the pathogenesis of degenerative joint diseases, including OA [12,13]. Alterations in the normal function of synoviocytes type B are related to OA, since pro-inflammatory cytokines released by the inflamed synovium shift chondrocyte activity to produce degradative enzymes, breaking down the cartilage and inhibiting tissue repair and regeneration [14]. 

Current evidence suggests that exercise is effective in enhancing musculoskeletal strength and power capacity, balance, range of motion, body coordination, functional joint stability, improves physical and psychological conditions, and decreases morbidity and pain in patients with OA [15,16]. In this way, muscles around the affected joints become stronger, bone loss and joint swelling decrease, and stiffness and pain improve thanks to a better lubrication of the joint cartilage [17]. However, the mechanisms mediating these effects are still not well understood. Since synoviocyte dysfunction is related to OA pathogenesis, due to the production by these cells of a number of enzymes and cytokines/chemokines that mediate tissue damage and inflammation, we hypothesized that moderate physical activity (MPA) might rescue type B synoviocyte function, as already reported for cartilage and muscle tissues [18], opening new perspectives for the prevention or treatment of OA.

Up-to-date scientific research on the biology of cartilages has been focused on finding a possible solution to treat advanced OA, either through cellular therapy, tissue engineering and other clinical methods, but few studies have dedicated their attention to OA prevention. When considering all of these aspects, and the multifactorial nature of OA in relation to hypomobility (aging, obesity, joint traumas, discordant joints, meniscal injuries, joint dislocation, ligamentous lesions, and so on...) and our previous scientific evidence [3,15,17], the purpose of this morphological study is to assess the influence of synovium alterations in the OA onset and to evaluate the effects of MPA on this tissue, suggesting MPA as a biological key for OA prevention, based on its effects as a natural anti-inflammatory remedy that also enhances joint lubrication.

For this purpose, we investigated synthesis and secretory activity of the synovium, specifically in relationship to the expression of OA biomarkers, such as IL-1β, IL-6, TNF-α, and MMP-13, and anti-inflammatory and chondroprotective markers, such as IL-4, IL-10, and lubricin, in OA-induced rats that were subjected to MPA.

## 2. Results

### 2.1. Histology

**Figure 1:**
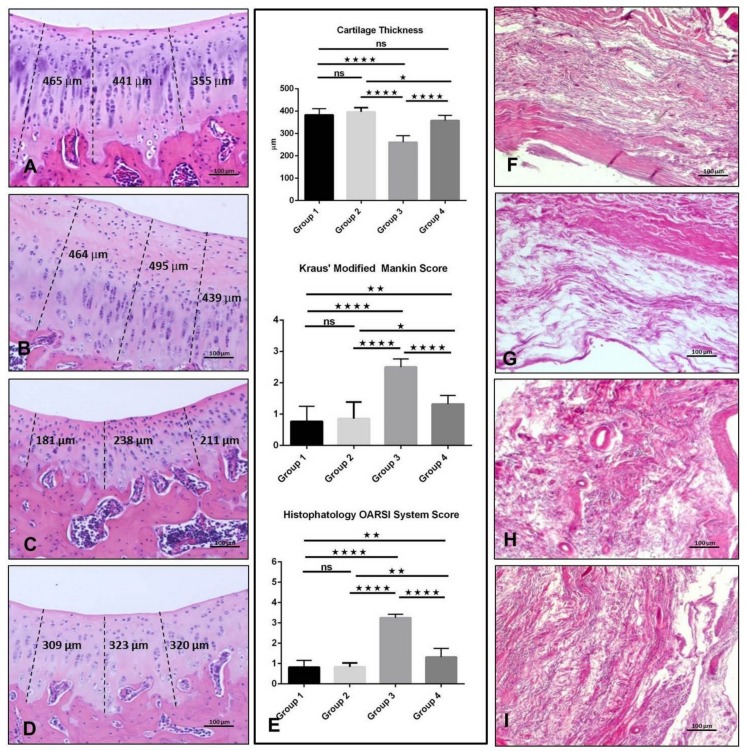
Histological evaluation and histomorphometric analysis of articular cartilage and synovium by Hematoxylin & Eosin (H&E) staining. (**A**) Group 1. Cells flat and small in the superficial zone; chondrocytes in columns in the middle and deep zone; tidemark evident; cartilage thickness 383.0 ± 76.76 µm. (**B**) Group 2. Numerous and small cells in the superficial zone; chondrocytes in columns in the middle and deep zone; tidemark evident; cartilage thickness 396.5 ± 61.68 µm. (**C**) Group 3. Articular cartilage showing structural alterations in the superficial and the middle zones and chondrocytes poorly organized in columns in the intermediate and deep zone; cartilage thickness 260.7 ± 62.34 µm. (**D**) Group 4. Articular cartilage showing only a slight reduction of the total thickness 357.9 ± 61.88 µm and a better general tissue preservation. (**E**) Graphs representing measures of the cartilage thickness, the Kraus’ Modified Mankin Score and the Histopathology OARSI System identified among groups. Results were presented as the mean ± SD. ANOVA was used to evaluate the significance of the results. * *p* < 0.05; ** *p* < 0.01; **** *p* < 0.0001; ns, not significant. For details, see the text. (**F**) H&E staining of synovium of Group 1. G H&E staining of synovium of Group 2. H H&E staining of synovium of Group 3. I H&E staining of synovium of Group 4. No histological alterations were evidenced in the synovium area of all groups. (**A**–**D,F**–**I**): Objective lens, 10×; scale bars: 100 µm.

Hematoxylin & Eosin (H&E) staining in articular cartilage samples was used to verify the onset of experimentally induced early OA through histomorphometric analyses detailed below. In Group 1 and 2, articular cartilage showed normal cytoarchitecture. Cells appeared flat and small in the superficial zone; chondrocytes displayed a columnar organization in the middle and deep zone; the tidemark was evident; cartilage thickness measured, respectively, 383.0 ± 76.76 and 396.5 ± 61.68 µm, with no statistical difference between them (*p* ≥ 0.05, ns = not significant) (Figure 1A,B,E). Group 3 showed signs of moderate OA. In fact, articular cartilage presented with structural alterations in the superficial and middle zones and chondrocytes were poorly organized in columns in the intermediate and deep zones; moreover, a reduction of thickness was observed (260.7 ± 62.34 µm) and the difference was statistically significant when compared with both Group 1 and 2 (*p* < 0.0001) (Figure 1C,E). In Group 4, better general tissue preservation was observed in comparison with Group 3, where the articular cartilage showed a slight but not statistically significant reduction in thickness (357.9 ± 61.88 µm) when compared with Group 1 (*p* ≥ 0.05, ns). The cartilage also showed a reduced number of cells (Figure 1D,E). From the histochemical analyses (H&E stainings) of synovium samples, synovium from neither group manifested any alteration, as expected (Figure 1F–I).

### 2.2. Histomorphometric Analyses

The histomorphometric analyses on articular cartilage samples were conducted to verify the onset of induced early OA. Histomorphometric parameters that were performed in Group 1 confirmed that there were no obvious signs of cartilage degeneration, with animals showing an intact and normal cartilage structure. Moreover, the Kraus’ Modified Mankin Score was 0.76 ± 0.87 and Histopathology OARSI System Score was 0.82 ± 0.76. Group 2 also showed no signs of cartilage degeneration, with a Kraus’ Modified Mankin Score of 0.85 ± 0.90 and a Histopathology OARSI System Score of 0.83 ± 0.73. Conversely, animals from Group 3 demonstrated pathological changes in the cartilage, with Kraus’ Modified Mankin Score of 2.5 ± 0.83, and Histopathology OARSI System Score of 3.25 ± 0.71. Group 4 showed a better cartilage preservation as compared to Group 3, with a Kraus’ Modified Mankin Score of 1.32 ± 0.76, and a Histopathology OARSI System Score of 1.32 ± 0.78 (Figure 1E). Statistical analyses highlighted that, for both scores, there was a significant difference among groups. In particular: Group 1 vs. Group 2, (*p* ≥ 0.05, ns) both in Kraus’ Modified Mankin Score and Histopathology OARSI System Score; Group 1 vs. Group 3, *p* < 0.0001 both in Kraus’ Modified Mankin Score and Histopathology OARSI System Score; Group 1 vs. Group 4 had a *p* = 0.0018 in Kraus’ Modified Mankin Score and a *p* = 0.0025 in Histopathology OARSI System Score; Group 2 vs. Group 3, had a *p* < 0.0001 both in Kraus’ Modified Mankin Score and in Histopathology OARSI System Score; Group 2 vs. Group 4, had a *p* = 0.0130 in Kraus’ Modified Mankin Score and a *p* = 0.0038 in Histopathology OARSI System Score; Group 3 vs. Group 4, *p* < 0.0001 in Kraus’ Modified Mankin Score and also in Histopathology OARSI System Score (Figure 1E). Inter-observer reliability among the five observers for the Mankin system showed a similar good intra-class correlation coefficient (ICC > 0.92) as for the OARSI system (ICC > 0.90). Repeated scoring by three of the five investigators resulted in a very good agreement (ICC > 0.94). Data are presented as the mean ± SD.

### 2.3. Immunohistochemistry (IHC) and Statistical Analysis

Different patterns of immunopositive cells in the sets of specimens were observed (Table 1).

**Figure 2:**
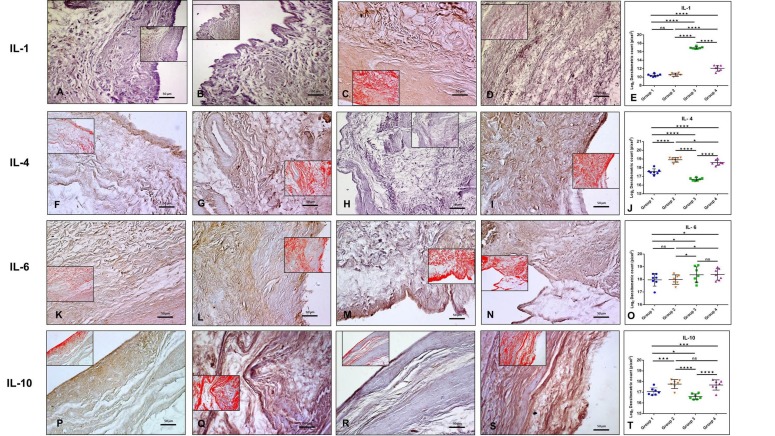
(**A**–**E**): IL-1β immunohistochemistry. (**A**) IL-1β immunolabeling was very weak in the synovium of Group 1; (**B**) IL-1β immunolabeling was very weak in the synovium of Group 2; (**C**) IL-1β immunolabeling was moderate in the synovium of Group 3; (**D**) IL-1β immunolabeling was weak in the synovial membrane of Group 4; (**E**) Graph representing the densitometric count (Log2 densitometric count − pixel^2^) of IL-1-immunolabeling identified among groups. For details, see the text. (**F**–**J**): IL-4 immunohistochemistry. (**F**) IL-4 immunolabeling was moderate in the synovium of Group 1; (**G**) IL-4 immunolabeling was strong in the synovium of Group 2; (**H**) IL-4 immunolabeling was weak in the synovium of Group 3; (**I**) IL-4 immunolabeling was strong in the synovial membrane of Group 4; (**J**) Graph representing the densitometric count (Log2 densitometric count − pixel^2^) of IL-4-immunolabeling identified among groups. For details, see the text. (**K**–**O**): IL-6 immunohistochemistry. (**K**) IL-6 immunolabeling was moderate in the synovium of Group 1; (**L**) IL-6 immunolabeling was strong in the synovium of Group 2; (**M**) IL-6 immunolabeling was very strong in the synovium of Group 3; (**N**) IL-6 immunolabeling was very strong in the synovial membrane of Group 4; (**O**) Graph representing the densitometric count (Log2 densitometric count − pixel^2^) of IL-6-immunolabeling identified among groups. For details, see the text. (**P**–**T**): IL-10 immunohistochemistry. (**P**) IL-10 immunolabeling was moderate in the synovium of Group 1; (**Q**) IL-10 immunolabeling was strong in the synovium of Group 2; (**R**) IL-10 immunolabeling was weak in the synovium of Group 3; (**S**) IL-10 immunolabeling was strong in the synovial membrane of Group 4; and, (**T**) Graph representing the densitometric count (Log2 densitometric count − pixel^2^) of IL-10-immunolabeling identified among groups. For details, see the text. In inserts are the image analyses by the software in which red color represents immunolabeling. (**A**–**D**,**F**–**I**,**K**–**N**,**P**–**S**): Objective lens, 20×; scale bars: 50 µm. Results were presented as the mean ± SD. ANOVA was used to evaluate the significance of the results. * *p* < 0.05; ** *p* < 0.01; *** *p* < 0.001; **** *p* < 0.0001; ns, not significant.

**Figure 3:**
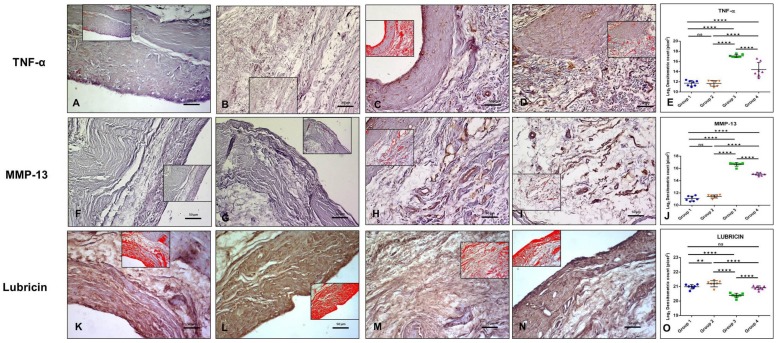
(**A**–**E**): TNF-α immunohistochemistry. (**A**) TNF-α immunolabeling was weak in Group 1; (**B**) TNF-α immunolabeling was weak in Group 2; (**C**) TNF-α-immunostaining was strong in Group 3; (**D**) TNF-α-immunostaining was weak in Group 4; (**E**) Graph representing the densitometric count (Log2 densitometric count − pixel^2^) of TNF-α-immunolabeling identified among groups. For details, see the text. (**F**–**J**): MMP-13 immunohistochemistry. (**F**) In Group 1, MMP-13 was very weak; (**G**) In Group 2, MMP-13 was very weak; (**H**) MMP-13-immunostaining was strong in Group 3; (**I**) MMP-13-immunostaining was weak in Group 4; (**J**) Graph representing the densitometric count (Log2 densitometric count − pixel^2^) of MMP-13-immunolabeling identified among groups. For details, see the text. (**K**–**O**): Lubricin immunohistochemistry. (**K**) Lubricin immunolabeling was strong in the synovium of Group 1; (**L**) Lubricin immunolabeling was very strong in the synovium of Group 2; (**M**) Lubricin immunolabeling was moderate in Group 3; (**N**) Lubricin immunolabeling was strong in the synovial membrane of Group 4; and, (**O**) Graph representing the densitometric count (Log2 densitometric count − pixel^2^) of Lubricin-immunolabeling identified among groups. For details, see the text. In inserts are the image analyses by the software in which red color represents immunolabeling. (**A**–**D**,**F**–**I**,**K**–**N**): Objective lens, 20×; scale bars: 50 µm. Results were presented as the mean ± SD. ANOVA was used to evaluate the significance of the results. ** *p* < 0.01; **** *p* < 0.0001; ns, not significant.

• IL-1β immunolabeling was very weak in the synovium of both Group 1 and 2 (ES = 0; IS = 1) (Figure 2A,B). IL-1β immunolabeling was moderate (ES = ++; IS = 2) in the synovium of Group 3 (Figure 2C). IL-1β immunolabeling was weak (ES = +; IS = 1) in the synovial membrane of Group 4 (Figure 2D).
• IL-4 immunolabeling was moderate in the synovium of Group 1 (ES = +; IS = 2) (Figure 2F). IL-4-immunostaining was weak (ES = +; IS = 1) in the synovium of Group 3 (Figure 2H) and it was strong in the synovial membrane of both Group 2 (ES = ++; IS = 3) (Figure 2G) and Group 4 (ES = ++; IS = 3) (Figure 2I).
• IL-6 immunolabeling was moderate in the synovium of Group 1 (ES = +; IS = 2) (Figure 2K). IL-6-immunostaining was strong (ES = +; IS = 3) in the synovium of Group 2 (Figure 2L). IL-6-immunostaining was very strong (ES = +++; IS = 4) in the synovium of both Group 3 (Figure 2M) and Group 4 (Figure 2N).
• IL-10 immunolabeling was moderate in the synovium of Group 1 (ES = ++; IS = 2) (Figure 2P). IL-10-immunostaining was weak (ES = +; IS = 1) in the synovium of Group 3 (Figure 2R) and it was strong (ES = +++; IS = 3) in the synovial membrane of both Group 2 and 4 (Figure 2Q,S).
• TNF-α immunolabeling was very weak in the synovium of both Group 1 and 2 (ES = 0; IS = 1) (Figure 3A,B). On the contrary, TNF-α-immunostaining was strong (ES = +++; IS = 3) in the synovium of Group 3 (Figure 3C) and weak (ES = +; IS = 1) in Group 4 (Figure 3D).
• MMP-13 immunolabeling was very weak in the synovium of both Group 1 and 2 (ES = 0; IS = 1) (Figure 3F,G). MMP-13-immunostaining was strong (ES = ++; IS = 3) in the synovium of Group 3 (Figure 3H) and it was weak (ES = +; IS = 1) in Group 4 (Figure 3I).
• Lubricin immunolabeling was strong in the synovium of both in Group 1 and 4 (ES = +++; IS = 3) (Figure 3K,N). It was very strong (ES = ++++; IS = 4) in the synovium of Group 2 (Figure 3L) and moderate (ES = ++; IS = 2) in the synovium of Group 3 (Figure 3M).

The above-mentioned results were confirmed by densitometric count (pixel), as obtained through image analysis by AxioVision software and statistical analysis, in which a statistically significant difference among groups was highlighted and in particular:

• IL-1β immunolabeling was much higher in Group 3 (16.92 ± 1.077) with respect to Group 1 (10.43 ± 1.139), Group 2 (10.56 ± 0.97), and Group 4 (12.08 ± 1.225). Group 1 and 2 had similar values (*p* ≥ 0.05, ns), whereas there was a statistically significant difference among groups (Groups 1 and 2 vs. 3, *p* < 0.0001; Groups 1 and 2 vs. 4, *p* < 0.0001; Group 3 vs. Group 4, *p* < 0.0001 (Figure 2E).
• IL-4 immunolabeling was higher both in Group 2 and 4 (respectively, 18.91 ± 0.768 and 18.59 ± 0.632) when compared to both Group 1 (17.55 ± 0.562) and Group 3 (16.67 ± 0.375). There was a statistically significant difference between Group 1 vs. Group 2, *p* < 0.0001; Group 1 vs. Group 3, *p* < 0.0001; Group 1 vs. Group 4, *p* < 0.0001; Group 2 vs. Group 3, *p* < 0.0001; Group 2 vs. Group 4, *p* = 0.0196; Group 3 vs. Group 4, *p* < 0.0001, (Figure 2J).
• IL-6 immunolabeling manifested a statistically significant difference between Group 1 (17.96 ± 0.786) vs. Group 3 (18.36 ± 1.106) (*p* = 0.0321) and Group 1 vs. Group 4 (18.39 ± 0.829) (*p* = 0.0188) with a higher IL-6 expression in both Group 3 and 4. Also, the differences between Group 2 (17.99 ± 0.638) vs. Group 3 and Group 2 vs. Group 4 were statistically significant, respectively *p* = 0.0468 and 0.0281. Conversely, the difference between Group 1 vs. Group 2 and between Group 3 vs. Group 4 was not statistically significant (*p* ≥ 0.05, ns) (Figure 2O).
• IL-10 immunolabeling was evident and similar in both Group 2 (17.76 ± 0.953) and Group 4 (17.68 ± 0.979) (*p* = ns). A statistically significant difference was evident among all groups and in particular: Group 1 (17.07 ± 0.817) vs. Group 2 had a *p* = 0.0001; Group 1 vs. Group 3 (16.59 ± 0.686) had a *p* = 0.0145; Group 1 vs. Group 4 had a *p* = 0.0009; Group 2 vs. Group 3 had a *p* < 0.0001; Group 3 vs. Group 4 had a *p* < 0.0001 (Figure 2T).
• TNF-α immunolabeling was higher in Group 3 (17.10 ± 0.608) when compared to all other groups, Group 1 (11.73 ± 0.968), Group 2 (11.69 ± 0.956) and Group 4 (14.39 ± 1.726) (Group 1 vs. Group 3, *p* < 0.0001; Group 2 vs. Group 3, *p* < 0.0001; Group 3 vs. Group 4, *p* < 0.0001). There was a statistically significant difference also between Group 1 vs. Group 4 (*p* < 0.0001); no statistical significance was found between Group 1 vs. Group 2 (*p* ≥ 0.05, ns), (Figure 3E).
• MMP-13-immunostaining was much higher in Group 3 (16.65 ± 0.664) when compared to Group 1 (11.09 ± 0.869) (*p* < 0.0001), Group 2 (11.42 ± 0.963) (*p* < 0.0001) and Group 4 (15.05 ± 0.646) (*p* < 0.0001). Furthermore, a statistically significant difference was shown also between Group 1 vs. Group 4 (*p* < 0.0001) and Group 2 vs. Group 4 (*p* < 0.0001). No statistically significant difference was found between Group 1 vs. Group 2 (*p* ≥ 0.05, ns) (Figure 3J).
• Lubricin immunolabeling in Group 3 (20.39 ± 0.275) was lower than in other groups. In particular: Group 1 (20.99 ± 0.226) vs. Group 3, *p* < 0.0001; Group 2 (21.19 ± 0.529) vs. Group 3, *p* < 0.0001; Group 3 vs. Group 4 (20.91 ± 0.267), *p* < 0.0001). Moreover, Group 1 vs. Group 2 had a statistically significant difference (*p* = 0.0066), whereas lubricin-immunostaining was similar in Group 1 and Group 4, hence showing no statistical difference (*p* ≥ 0.05, ns) (Figure 3O).

## 3. Discussion

**Figure 4:**
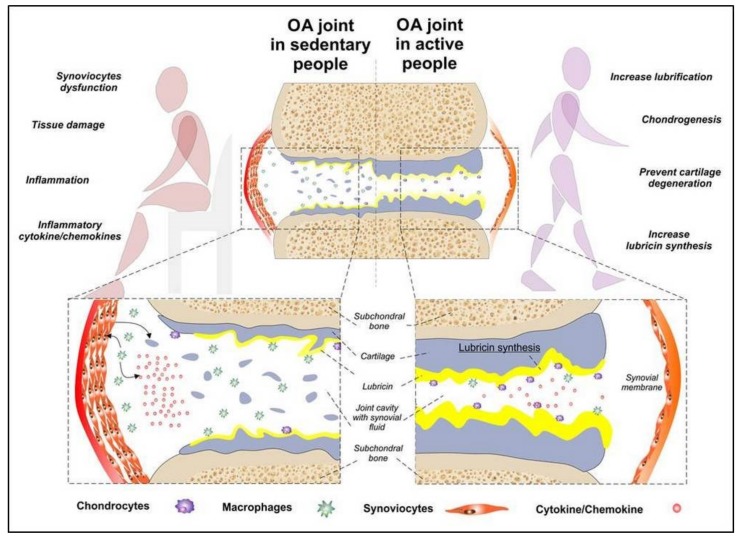
Graphical representation of a joint affected by moderate osteoarthritis (OA) in sedentary people and in active people with relative benefits and disadvantages. Pro-inflammatory cytokines play a key role in the pathogenesis of OA, by mediating the progressive degeneration of the articular cartilage within the joint. The deteriorating processes involve different types of cells including macrophages, chondrocytes and synoviocytes.

The purpose of this study was to investigate the possible influence of MPA on the expression of OA-related biomarkers as well as anti-inflammatory and chondroprotective markers by synoviocytes type B in OA rat model. As recommended by World Health Organization, MPA refers to the activity that requires energy of 3.0 to 5.9 times higher than that of the resting state [19]. The MPA-based approach may support joint tribology and synovial lubrication, leading to improved joint function and pain relief. Our hypothesis is that the adapted MPA/movement may counteract synoviocyte Type B dysfunction at the early stages of OA, impeding overt onset of OA. Potential physical non-pharmacologic treatment strategies at the initial stages of OA may reduce the pathological burden of the condition at its early stages and possibly postpone the need for joint replacement (Figure 4). 

In the present work, we used a well-established model to induce an early OA through the anterior cruciate ligament transection (ACLT) technique [20,21,22]. The ACLT model is a short-term surgically induced OA model that in this study has surely benefited from exercise. This model of early OA is different from chronic OA based on the long-term inflammation, where the disease develops over decades and it is often combined with other systemic conditions and comorbidities, such as sarcopenia [13,16]. However, as well known, if OA is not adequately treated at its prodromal stages by life style interventions, such as MPA and/or surgery or pharmacological therapy, then OA would become chronic due to the ongoing joint instability and consequent joint degeneration [20,21,22].

The results that were obtained in the present study on synovium, are in line with both our previous data and those of other authors on several joint tissues [15,16,17,20,21,22,23,24,25,26,27,28]. In this morphological study, we investigated the expression of some pro-inflammatory molecules (IL-1β, IL-6, TNF-α), OA-related enzymes (MMP-13), anti-inflammatory cytokines (IL-4, IL-10), and chondroprotective markers (lubricin) in synovium of OA-induced rats that were subjected to MPA. 

Firstly, in the ACLT-rat group (Group 3), the above-mentioned OA-related markers were significantly increased, while IL-4, IL-10, and lubricin expression were drastically decreased when compared with Groups 1 and 2, as expected. Lubricin is one of the major joint lubricants. It is a glycoprotein, as expressed by the chondrocytes from the superficial layer and by synoviocytes B and it has received considerable attention as a chondroprotective molecule [29,30,31,32]. The relationships between articular cartilage, boundary lubrication, and alterations in cartilage tissue after injury have not yet been clearly understood, but it emerges from literature that poor lubrication could predispose the articular cartilage to degeneration, thereby promoting the development of OA [31,33]. Joint immobilization, in OA patients, facilitates future damage and chondrocyte apoptosis, since it inhibits the release within the joint cavity of synovial fluid that represents the main source of nourishment for the cartilage, which is rich in glycosaminoglycans, such as hyaluronan, and proteoglycans, such as lubricin [15,34]. On the other hand, MPA with normal joint loading generates, through mechanical stimulation, a greater synovial fluid perfusion of the superficial layer of the cartilage, improving lubrication, chondrocyte homeostasis, and proliferation, whilst preventing cartilage degeneration [20]. In the present study, lubricin expression was significantly decreased in ACLT-rats group (Group 3) as compared with Groups 1 and 2, which agrees with previous results from studies on OA articular cartilage chondrocytes [22,35], thus suggesting its important role as a chondroprotective agent. 

The results from our MPA experimental group (Group 4: ACLT-rats and MPA) highlighted the decreased expression of OA-related biomarkers (IL-1β, TNF-α, MMP-13) and the increased expression of chondroprotective ones (IL-4, IL-10, and lubricin) following physical activity, prompting on the beneficial effect of MPA on the synovium and, consequently, on cartilage preservation. In pathologic conditions, synoviocytes type A produce and secrete cathepsins, MMPs, and pro-inflammatory cytokines/chemokines into the extracellular matrix, triggering tissue damage and, in turn, resulting in OA onset [11,22]. Exercise therapy may decrease cytokines and related genes expression and inhibit inflammatory factors-mediated cartilage degradation, through the synthesis of IL-10 by synoviocytes type A, thus, effectively blocking cartilage damage [36,37]. Our results on MMP-13 expression in the synovium are in accordance with the above-mentioned literature, as Group 4 (ACLT-rats and MPA) displayed significantly reduced MMP-13 levels, whilst the IL-10 levels were significantly increased. 

Concerning the pro-inflammatory cytokine IL-6, we observed that its expression in Group 4 did not decrease, as we would expect, but it remained similar to Group 3 (ACLT osteoarthritic rats). During the initial phase of the inflammatory process, the production of IL-6 is likely to be due to the activity of type A synoviocytes. Subsequently, heightened IL-6 production might persist due to the action of type B synoviocytes, which are transformed into fibroblasts [38]. Our explanation is that moderate exercise cannot revert the cellular transformation of type B synoviocytes, but at most it can reduce/impede further transformations.

The role of IL-6 has been widely treated in the literature and there are no doubts regarding its pro-inflammatory function. It has been shown that it is promptly produced in response to tissue injuries and that its dysregulated synthesis has pathological consequences featured during chronic inflammatory states [39]. However, IL-6, in some cases, exerts protective effects [40,41]. Furthermore, during exercise, IL-6 is produced and released by contracting skeletal muscle fibers, exerting its effects in other organs of the body, as reported in literature [42], and for this reason, IL-6 is considered often referred to as a “myokine”. As a myokine, IL-6 stimulates the secretion into the circulation of anti-inflammatory cytokines, such as IL-1RA and IL-10, and inhibits the production of additional pro-inflammatory cytokines, including TNF-α by synoviocytes type A cells [34]. These other roles of IL-6 might, probably, explain the similar expression of this cytokine that we have seen in Group 4. Nevertheless, the role of IL-6 should be certainly further studied and clarified. The weakness of the present study was to evaluate only the morphological evidences based on the immunohistochemistry methods, to investigate the expression of OA-related biomarkers as well as anti-inflammatory and chondroprotective markers. Further studies are needed to confirm our morphological data with other relevant and sensitive techniques, such as quantitative RT-PCR, ELISA (or similar), and/or western blot.

In conclusion, the taboo that joints that are affected by OA should not be subjected to physical activity and must be absolutely disproved, as many international medical societies have recently recommended. The benefits arising from physical activity for the prophylaxis and treatment of a number of chronic conditions are renowned. For some chronic pathologies, well-planned exercise interventions and a healthy diet are at least as effective as drug therapy [43,44,45]. MPA in OA knee patients (walking, moderate running, swimming, riding a bike, and using elliptical machines) may protect joints against the damaging effects of excessively repetitive joint use and replace glycoproteins lost during periods of immobility, improving clinical benefits [46]. Moreover, as a non-surgical and non-pharmacological approach, MPA could re-establish normal synoviocyte function at the initial phases of OA, hence delaying the onset of overt OA and finally postponing the need for joint replacement. It needs, however, to report recent scientific data showing that moderate physical activity, in some cases and specific experimental conditions, does not protect against the development of OA [47]. Indeed, further studies are warranted to further validate our findings. Preservation of the joint health is critical for retaining independent living, a good health status, and quality of life. We hope that this contribution and theory may help readers and the scientific community to gain a better understanding of the importance of MPA in OA treatment. Exercise is an effective evidence-based medicine, and our findings have, at least in part, provided a proof-of-concept consisting of morphological, molecular, and biochemical evidence to support the statement that there is less risk in activity than in continuous inactivity.

## 4. Materials and Methods

### 4.1. Breeding, Housing of Animals and Experimental Design

For the purpose of these studies, we used thirty-two adult male Wistar rats (Charles River Laboratories, Milan, Italy), with an average body weight of 250 ± 20 g. Rats were housed in polycarbonate cages (cage dimensions: 10.25″W × 18.75″D × 8″H) at controlled temperature (24 °C) and humidity during the whole period of the research (14 weeks) with access to water and food ad libitum and 12 h light/dark cycle. Animals were divided into four groups: Group 1, control health rats (*n* = 8); Group 2: rats performing MPA (treadmill training exercise) (*n* = 8); Group 3: ACLT-rats with early OA (*n* = 8); and, Group 4: ACLT-rats with early OA performing MPA (treadmill training exercise) (*n* = 8).

**Figure 5:**
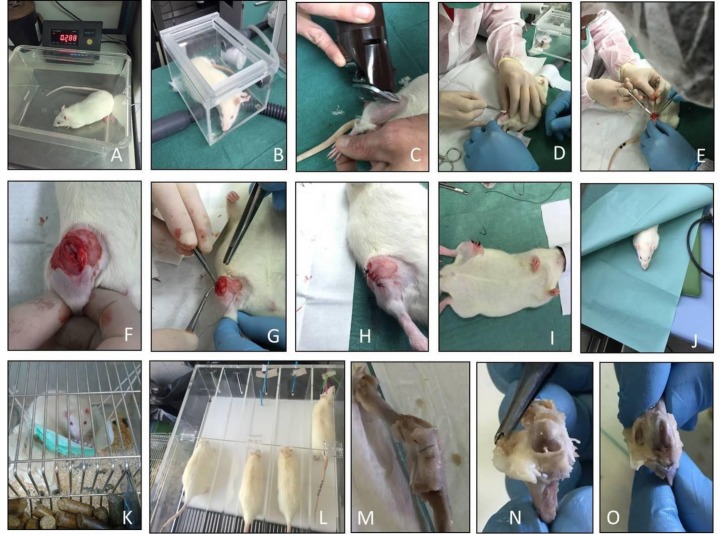
Surgical procedures for anterior cruciate ligament transection (ACLT) step by step. (**A**) Pre-operative examinations and weighing of the rat; (**B**) Anesthesia of the rat; (**C**) Shearing of the rat; (**D**) Cutting the skin of the rat; (**E**) Cutting the capsule of the rat; (**F**) The patella was displaced laterally to expose the anterior cruciate ligament, then, the anterior cruciate ligament was cut with surgical scissors; (**G**) The skin around the knee cap was vertically incised along the medial border of the knee cap; (**H**) The patella was then replaced back, and the fascia and skin were closed with a 3–0 polydioxanone suture; (**I**) The rat after the suture under anesthesia before awakening; (**J**) The rat in the heated bed before awakening from anesthesia; (**K**) After surgery, free cage movement without joint immobilization was permitted to the rat; (**L**) Rats from Group 3 during exercise on Treadmill; (**M**) Knee joint covered to capsule; (**N**) Capsule removal; and, (**O**) Knee joint without capsule.

Surgical procedures for anterior cruciate ligament transection (ACLT) were performed in accordance with the method that was previously described [19,20]. The ACLT surgery (Figure 5) procedure was made under total anesthesia to induce the OA model, 30 mg/kg Zoletil 100 + altadol 5 mg/kg + maintenance mixture of O_2_ and isoflurane 2–2.5%, (Vibrac, Milan, Italy). The anterior portion of the left hind limb was shaved with an electric clipper and cleaned with ethanol. The skin around the knee cap was vertically incised along the medial border of the knee cap. The patella was displaced laterally to expose the anterior cruciate ligament. Subsequently, the anterior cruciate ligament was cut with surgical scissors without injury to the cartilage of the tibia. The patella was then replaced back and the fascia and skin were closed with a 3-0 polydioxanone suture. A single dose of antibiotic Convenia^®^ 0.1 mL/kg, (Vibrac) cream was applied to avoid postoperative infection. After surgery, free cage movement without joint immobilization was permitted to all animals. The pre-operative examinations included physical and photographical examination.

At the end of the experiment, after the physical activity protocol, animals were humanely sacrificed by an intravenous lethal injection of anesthetic overdose using a mixture of Zoletil 100 (Virbac) at a dose of 80 mg/kg and DEXDOMITOR (Virbac) at a dose of 50 mg/kg. After euthanasia, the synovial membrane from knee joint and the articular cartilage were explanted, and the samples were used to perform histological and immunohistochemical evaluation in synovium and histomorphometric analysis in articular cartilage. All of the experiments were designed to minimize animal suffering and to use the minimum number of animals required to achieve a valid statistical evaluation according the principles of the 3R’s (replacement, reduction and refinement of animal use). All of the procedures conformed to the guidelines of the Institutional Animal Care and Use Committee (I.A.C.U.C.) at the Center for Advanced Preclinical In Vivo Research (CAPIR), University of Catania, and approved by Italian Ministry of Health, Protocol n. 2112015-PR of the 14.01.2015 (14 January 2015). The experiments were conducted in accordance with the European Community Council Directive (86/609/EEC) and the Italian Animal Protection Law (116/1992).

### 4.2. Treadmill Training Exercise

Following two weeks of acclimatization to the housing environment after ACLT, rats from Groups 2 and 4 were exposed to their respective exercise program on the treadmill (2Biological instrument, Varese, Italy), for 12 weeks [1,17]. The exercise (from mild to moderate) progressively built up to 25 min of treadmill training each day, three times per week at 20 meters/min (see Table 2 for details of the exercise program). The treadmill was inclined at 2° (between two and six degrees). A mild electric shock (0.2 mA) forced the rat to walk on the treadmill at a speed and inclination adapted to it. The shock serves to stimulate the rat to walk and to instruct it. The rat usually learns this activity in the first two minutes of exercise. This type of exercise is used to stimulate the muscles, joints, and bones in the work of flexion-extension of the limbs and to release more synovial fluid into the articular capsula. All electric shock data were acquired by Data acquisition software (2Biological instrument, Varese, Italy). Before and after exercise, rats were left free without immobilization in their cages. During the exercise, the possible suffering of the animal was evaluated. Potential rats discarded from the experiment: rats that exhibited difficulties in walking; and, rats that exceeded the number of 10 electric shocks (0.2 mA) without learning the work to be done on the treadmill. None of the rats presented any of these characteristics and thus no animals were excluded from the study.

### 4.3. Histology Analysis

Synovium and articular cartilage samples were fixed in 10% neutral buffered-formalin (Bio-Optica, Milan, Italy), following overnight washing and routinely embedded in paraffin, as previously described [48]. Samples were positioned in the cassettes after wax infiltration. A rotary manual microtome (Leica RM2235, Milan, Italy) was used to cut 4–5 μm thick sections from paraffin blocks that were mounted on silane-coated slides (Menzel-Gläser, Braunschweig, Germany) and stored at room temperature. After dewaxing in xylene, the slides were hydrated using graded ethanol and were stained by Hematoxylin & Eosin (H&E) staining for histological and histomorphometric evaluation, detecting possible structural alterations in tissues. The samples were examined with a Zeiss Axioplan light microscope (Carl Zeiss, Oberkochen, Germany) and a digital camera (AxioCam MRc5, Carl Zeiss) was used to take the pictures.

### 4.4. Histomorphometric Analysis

The femur explantation procedure and the subsequent cleaning of soft tissues were performed as previously described [21]. Samples from all rats (both medial and lateral femoral condyles of untreated and surgically treated animals) were used for the histomorphometric analysis. Histomorphometry was performed with image analysis, Kontron KS 300 software (Kontron Electronics, Eching bei Munchen, Germany) by three blinded investigators (two anatomical morphologists and one histologist). Evaluations were assumed to be correct if there were no statistically significant differences between the investigators. Seven fields randomly selected from each section were analysed. Multiple measurements of cartilage thickness in different points of cartilage were detected in all groups and the semi-quantitative grading criteria of macroscopic Kraus’ modified Mankin score [49] and microscopic histopathology OARSI system [50] were used. 

The Kraus’ modified Mankin score provides grades from 0 to 4: Grade 0, normal cartilage; Grade 1, minimal articular damage; Grade 2, articular cartilage damage affecting up to 30% of the articular surface; Grade 3, loss of up to 50% of the articular cartilage; Grade 4, severe loss of cartilage affecting more than 50% of the articular surface.

The Histopathology OARSI system provides grades from 0 to 6: Grade 0, normal articular cartilage; Grade 1, intact surface; Grade 2, surface discontinuity; Grade 3, vertical fissures extending into mid zone; Grade 4, erosion; Grade 5, denudation; Grade 6, deformation.

### 4.5. Immunohistochemistry (IHC) Analysis

Synovium samples were processed for immunohistochemical analysis, as previously described [51]. Briefly, after dewaxing in xylene, the slides were hydrated through graded ethanol and incubated for 30 min in 0.3% H2O2/methanol to quench endogenous peroxidase activity and then rinsed for 20 min with phosphate-buffered saline (PBS; Bio-Optica). The sections were then heated (5 min × 3) in capped polypropylene slide-holders with citrate buffer (10 mM citric acid, 0.05% Tween 20, pH 6.0; Bio-Optica), using a microwave oven (750 W) to unmask antigenic sites. The blocking step to prevent non-specific binding of the antibody was performed before application of the primary antibody with 5% bovine serum albumin (BSA, Sigma, Milan, Italy) in PBS for 1 h in a moist chamber. After blocking, the sections were incubated overnight at 4°C with rabbit polyclonal anti-IL-1β (ab9787; Abcam), diluted 1:100 in PBS (Bio-Optica); rabbit polyclonal anti-IL-4 (ab9622; Abcam), diluted 1:100 in PBS (Bio-Optica); goat polyclonal anti-IL-6 antibody (sc-1265; Santa Cruz Biotechnology Inc., Dallas, TX, USA) diluted 1:100 in PBS (Bio-Optica); rabbit polyclonal anti-IL-10 (ab34843; Abcam), diluted 1:100 in PBS (Bio-Optica); mouse monoclonal anti-TNF-α antibody (ab199013; Abcam ) diluted 1:100 in PBS (Bio-Optica); mouse monoclonal anti-MMP-13 antibody (sc-81547; Santa Cruz Biotechnology Inc.) diluted 1:100 in PBS (Bio-Optica); rabbit polyclonal anti-lubricin antibody (ab28484; Abcam), diluted 1:100 in PBS (Bio-Optica). Immune complexes were then treated with a biotinylated link antibody (HRP-conjugated were used as secondary antibodies) and were then detected with peroxidase labeled streptavin, both incubated for 10 min at room temperature (LSAB+ System-HRP, K0690, Dako, Glostrup, Denmark). The immunoreaction was visualized by incubating the sections for 2 min in a 0.1% 3,3′-diaminobenzidine and 0.02% hydrogen peroxide solution (DAB substrate Chromogen System; Dako). The samples were lightly counterstained with Mayer’s Hematoxylin (Histolab Products AB, Goteborg, Sweden) mounted in GVA mount (Zymed, Laboratories Inc., San Francisco, CA, USA) and observed with an Axioplan Zeiss light microscope (Carl Zeiss) and photographed with a digital camera (AxioCam MRc5, Carl Zeiss).

### 4.6. Evaluation of Immunohistochemistry

The IL-1β-, IL-4-, IL-6-, IL-10-, TNF-α-, MMP-13-, and lubricin-staining status was identified as either negative or positive. As previously described, immunohistochemical staining was defined positive if brown chromogens were detected on the edge of the hematoxylin-stained cell nucleus, within the cytoplasm or in the membrane [52]. Light microscopy was used to evaluate stain intensity and the percentage of immunopositive cells. Intensity of staining (IS) was evaluated on a four grades scale (0–4), as following: no detectable staining = 0, weak staining = 1, moderate staining = 2, strong staining = 3, and very strong staining = 4. Three investigators (2 anatomical morphologists and one histologist) independently evaluated the percentage of antibodies immunopositive cells through the five categories of Extent Score (ES): <5% (0); 5–30% (+); 31–50% (++); 51–75% (+++); and, >75% (++++). Counting was performed under Zeiss Axioplan light microscope at 200× magnification. If disputes concerning the interpretation occurred, the case was revised to reach a unanimous agreement, as previously described [53]. A digital camera (Canon, Tokyo Japan) at 20×, 40× and 60× magnifications was used to take digital pictures. In this study, positive controls, consisting of rat cartilage tissue, and negative control sections, which were treated with PBS without the primary antibody, were performed to test the specific reaction of primary antibodies used at a protein level. Positive immunolabeling for antibodies were nuclear/cytoplasmic.

### 4.7. Computerized Densitometric Measurements and Image Analysis

Image analysis software (AxioVision Release 4.8.2-SP2 Software, Carl Zeiss Microscopy GmbH, Jena, Germany), which quantifies the level of staining of positive anti-IL-1β, anti-IL-4, anti-IL-6, anti-IL-10, anti-TNF-α, anti-MMP-13, and anti-lubricin antibodies immunolabelling, was used to calculate the densitometric count (pixel^2^) in seven fields, area of which was about 150.000 µm^2^, randomly selected from each section. Digital micrographs were taken using the Zeiss Axioplan light microscope (Carl Zeiss, using objective lens of magnification 20× i.e., total magnification 200×) fitted with a digital camera (AxioCam MRc5, Carl Zeiss). Three blinded investigators (two anatomical morphologists and one histologist) made the evaluations that were assumed to be correct if values have not statistically significant difference [54]. If disputes concerning interpretation occurred, unanimous agreement was reached after sample re-evaluation. 

### 4.8. Statistical Analysis

Statistical analysis was performed using GraphPad Instat^®^ Biostatistics version 3.0 software (GraphPad Software, Inc. La Jolla, CA, USA). Analysis of variance (one-way ANOVA—Tukey’s multiple comparisons test) was used for comparison between more than two groups; Unpaired *t* test with Welch’s correction was used for comparison between two groups. *p*-values of less than 0.05 were considered to be statistically significant (* *p* < 0.05; ** *p* < 0.01; *** *p* < 0.001; **** *p* < 0.0001 and ns: not significant). Data are presented as the mean ± SD.

## Figures and Tables

**Table 1:** Evaluation of IL-1β-, IL-4-, IL-6-, IL-10-, TNF-α-, MMP-13-, and lubricin-immunostaining. Intensity of staining (IS) was graded on a scale of 0–4, based on the following criteria: No detectable staining (0), weak staining (1), moderate staining (2), strong staining (3), and very strong staining (4). The percentage of immunopositive cells (Extent Score = ES) was independently evaluated by three investigators (two anatomical morphologists and one histologist) and ranked according to the following criterium: <5% (0); 5–30% (+); 31–50% (++); 51–75% (+++), and >75% (++++).

Group	IL-1β	IL-4	IL-6	IL-10	TNF-α	MMP-13	Lubricin
1	ES = 0 IS = 1	ES = + IS = 2	ES = + IS = 2	ES = ++ IS = 2	ES = 0 IS = 1	ES = 0 IS = 1	ES = +++ IS = 3
2	ES = 0 IS = 1	ES = ++ IS = 3	ES = + IS = 3	ES = +++ IS = 3	ES = 0 IS = 1	ES = 0 IS = 1	ES = ++++ IS = 4
3	ES = ++ IS = 2	ES = + IS = 1	ES = +++ IS = 4	ES = + IS = 1	ES = +++ IS = 3	ES = ++ IS = 3	ES = ++ IS = 2
4	ES = + IS = 1	ES = ++ IS = 3	ES = +++ IS = 4	ES = +++ IS = 3	ES = + IS = 1	ES = + IS = 1	ES = +++ IS = 3

**Table 2:** Twelve-week treadmill training program (mild to moderate physical activity).

Week	Speed (m/min)	Session/Week	Session/Day	Duration (min)
1	5	3	1	5
2	10	3	1	10
3–6	15	3	1	15
7–12	20	3	1	25
